# Bimanual Intravenous Needle Insertion Simulation Using Nonhomogeneous Haptic Device Integrated into Mixed Reality

**DOI:** 10.3390/s23156697

**Published:** 2023-07-26

**Authors:** Jin Woo Kim, Jeremy Jarzembak, Kwangtaek Kim

**Affiliations:** 1 Computer Science, Kent State University, 800 E Summit St, Kent, OH 44240, USA; jkim80@kent.edu; 2 College of Nursing, Kent State University, 800 E Summit St, Kent, OH 44240, USA; jjarzemb@kent.edu

**Keywords:** IV needle insertion simulation, dual haptic rendering, mixed reality, bimanual haptic interface, haptic-glove-based interaction, hand motor skill training, nursing education

## Abstract

In this study, we developed a new haptic–mixed reality intravenous (HMR-IV) needle insertion simulation system, providing a bimanual haptic interface integrated into a mixed reality system with programmable variabilities considering real clinical environments. The system was designed for nursing students or healthcare professionals to practice IV needle insertion into a virtual arm with unlimited attempts under various changing insertion conditions (e.g., skin: color, texture, stiffness, friction; vein: size, shape, location depth, stiffness, friction). To achieve accurate hand–eye coordination under dynamic mixed reality scenarios, two different haptic devices (Dexmo and Geomagic Touch) and a standalone mixed reality system (HoloLens 2) were integrated and synchronized through multistep calibration for different coordinate systems (real world, virtual world, mixed reality world, haptic interface world, HoloLens camera). In addition, force-profile-based haptic rendering proposed in this study was able to successfully mimic the real tactile feeling of IV needle insertion. Further, a global hand-tracking method, combining two depth sensors (HoloLens and Leap Motion), was developed to accurately track a haptic glove and simulate grasping a virtual hand with force feedback. We conducted an evaluation study with 20 participants (9 experts and 11 novices) to measure the usability of the HMR-IV simulation system with user performance under various insertion conditions. The quantitative results from our own metric and qualitative results from the NASA Task Load Index demonstrate the usability of our system.

## 1. Introduction

One of the most common invasive procedures patients experience in the hospital setting is the insertion of an intravenous catheter (IVC) [1]. Infusion therapy utilizing intravenous catheters provides a route to administer life-sustaining fluids, electrolyte replacement, and pharmacological agents and provides a means to extract blood for testing and diagnostic purposes. Although there are numerous risks associated with accessing the venous system directly, it is often the best and/or only route of choice [2]. Unfortunately, not all IVC insertions are successful, especially on the first attempt, requiring additional attempts that are stressful and painful for the patient. Venipuncture skills are among the most challenging for the novice nurse to master when they are in training and transitioning into practice. When intravenous (IV) access is unsuccessful, patients experience delays in treatment and unnecessary discomfort. Poor success rates are due to confidence issues, improper angle of insertion, and lack of opportunities. These factors contribute to increasing stress on new nurses and poor patient outcomes [1]. Educational opportunities to train IVC skills typically consist of practicing on plastic manikins, providing a poor replacement for the realism required to achieve mastery. In addition, teaching this skill requires many consumable products and costly medical devices, e.g., IV catheters that are single-use only and manikin arms that provide users with easy recognition of site locations through repeated attempts in the same vein with no realism or variability. Therefore, creation of a realistic, repeatable, cost-effective, and variability-controllable device is needed to improve the psychomotor skill development necessary for enhancing learning to achieve mastery. Many studies have shown the effectiveness of haptic feedback with virtual reality [3,4,5,6,7,8] or augmented reality [9,10,11,12,13,14] for improving insertion training. However, existing haptic simulators have limitations, including lack of variability, lack of realism, and featuring only single modalities, for development of psychomotor skills needed for proper IV insertion.

To improve psychomotor skill acquisition, this study devised a new approach by utilizing cutting-edge technology to meet these challenges. First, we developed a bimanual haptic mixed reality IV insertion simulation that allowed for a variety of different insertion conditions. Second, we created a force-profile-based haptic rendering algorithm that provides realistic haptic feedback to the user while inserting the needle into the virtual patient’s vein. Third, we improved haptic glove tracking using a hand-tracking sensor. Fourth, we calibrated the haptic and mixed reality system with a calibration box using the HoloLens camera sensor. Fifth, we conducted an experiment to verify the realism of the system and calibrated it to create a suitable environment for needle insertion. Finally, we conducted an experiment using human subjects to measure the usability of the system based on user performance using our own metric and the NASA Task Load Index (TLX) [15].

The remainder of this article is organized as follows. In Section 2, we present related studies, including needle insertion simulation for surgical procedures or biomedical applications. In Section 3, we describe development of a bimodal haptic simulation system using two non-homogenous haptic devices integrated with mixed reality. Section 4 presents the experiments conducted to evaluate the developed system. The results are reported in Section 5. Section 6 and Section 7 provide the discussion and conclusion, respectively.

## 2. Related Work

Various approaches using medical simulations with haptic feedback in a mixed reality (MR) environment take advantage of their realism with hand–eye coordination. Si et al. [16] developed a bimanual neurosurgical simulation in a mixed reality environment with two Geomagic Touch devices. A holographic brain was created and registered to a 3D-printed skull that showed small registration errors, which were 4.61 mm from nine fiducial points. However, usability of the system was not proven. Guo et al. [11] also implemented a bimanual surgical training system for the renal biopsy procedure using two Geomagic Touch devices that demonstrated improvement of trainees’ puncture ability after training. Several studies have also shown the benefits of augmented reality using HoloLens in needle insertion into a target organ [12], epidural needle insertion simulation [13], and psychomotor skills development for Veress needle insertion [14]. However, none of the existing MR simulations are aimed at simulating IV needle insertion as well as providing bimodal haptic feedback using two different devices (haptic glove and haptic stylus). Furthermore, very little is known about how to accurately collocate different modalities in real-time for realistic needle insertion simulation.

Differing from MR simulations, initial studies of needle insertion simulations showed the potential for or possibility of using a haptic stylus device in virtual reality (VR) and augmented reality (AR) systems without a head-mounted display (HMD). Wang et al. [3] developed a real-time haptic interactive 3D brachytherapy simulation of needle insertion. The system is equipped with a deformable soft tissue model using the 3D ChainMail method. However, this deformation model is not suitable for veins when it comes to needle insertion. Ni et al. [4] developed an ultrasound-guided biopsy training system to simulate a stitching task on ultrasound imagery using overlaid ultrasound volumes based on a 3D scale-invariant feature transformation algorithm. Similar to our system, their force model contains different layers such as skin, muscle, adipose, and liver. However, there is a space gap between the haptic workspace and display, which can create errors in spatial coordinates. Shin et al. [5] developed a haptic injection simulator that is limited to two degrees of freedom (DOF) by designing a microprocessor-based needle actuator. This has the advantage of eliminating loss or distortion of haptic feedback compared to other complex haptic devices using belts or gears. Coles et al. [9] developed PalpSim, an augmented reality (LCD screen display) bimanual simulation system for training femoral palpation and needle insertion in interventional radiology procedures. For the palpation step, two Falcon (haptic) devices are linked together to provide 5 DOF feedback combined with a custom-built hydraulic interface. For the needle insertion component, a PHANToM Omni haptic device was employed using a modified interface that allowed for using a real needle during insertion to replicate the necessary force feedback, combined with augmented reality, a virtual patient, virtual blood flow, and the user’s hands displayed through an LCD monitor. However, the complexity of the required hardware distracts from the realism. Xia et al. [6] presented a low-cost approach for image-based virtual haptic venipuncture simulation with multilayer soft tissue using a PHANToM Omni device. The intuitive visibility using a real image was clear. However, the uncalibrated scale between the screen and haptic device and the depth recognition of the 2D image were limitations. Sutherland et al. [7] developed a prototype of an augmented-reality-based haptic simulation for spinal needle insertion training utilizing an overlaid cutaway of the patient’s anatomy onto the torso of the manikin with an optical camera. The overlayed CT image of the manikin looked very accurate. However, the accuracy of registration was not provided, and the generated display was not realistic. Fortmeier et al. [10] developed a visuo-haptic framework and methods of direct visuo-haptic 4D volume rendering for a virtual patient model whose surface is continuously deformed by breathing motion, providing more realism than a static object. Haptic feedback using the PHANToM Premium was integrated into the deformable visual volume to simulate haptic palpation and needle insertion. However, the computation time increases depending on the number of voxels. To maintain stable rendering, deformable motion was not included in our system. Overall, despite the effort to provide more realistic experiences, these simulations still had gaps compared to real needle insertion environments. Vaughan et al. [17] created a paramedic virtual reality training simulator that integrates Oculus Rift with a 3 DOF PHANToM Omni device to simulate the needle cricothyroidotomy procedure. Although the visual of the simulation provides more realism through HMD, the paper does not provide enough details about the algorithm for haptic feedback. Ortegon et al. [18] developed a multimodal virtual reality medical simulation prototype for epidural procedure practices using three different haptic and VR prototypes: Oculus Rift; Touch 3D, a mobile VR headset with two Falcon devices modified to provide one knob interface; and Oculus Rift with Geomagic Touch. The comparison of usability between different HMDs was meaningful, but the details of the needle insertion process were not stated. Based on our literature review, none of the existing studies have shown the feasibility of a bimanual haptic interface using two different haptic devices, a haptic glove (Dexmo) and a stylus haptic device (Geomagic Touch), combined with HoloLens 2 to simulate a realistic IV needle insertion procedure with variabilities that mimic a real clinical environment.

## 3. Development of Bimanual Haptic–Mixed Reality IV Needle Insertion Simulation with Variability

**Figure 1:**
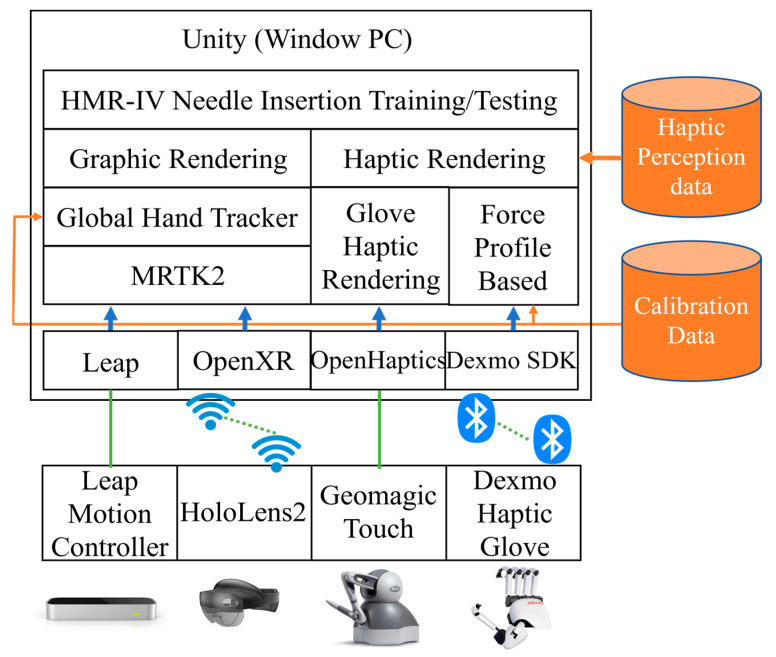
The architecture of the HMR-IV needle insertion simulation system proposed and developed in this study. The data used are represented by orange lines, while the open libraries used for implementation are shown by blue arrows. The wired connections are indicated by green solid lines, and the wireless connections are represented by dotted lines.

**Figure 2:**
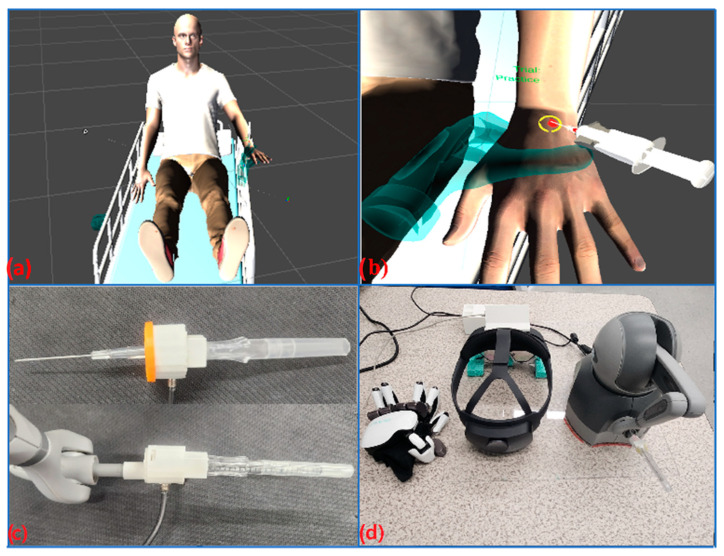
HMR-IV simulation system developed in this study: (**a**) a virtual patient, (**b**) a screenshot of graphic needle insertion simulation using two hands (the left hand for stabilizing and the right hand for inserting a virtual needle), (**c**) a modified haptic needle interface (a real IV catheter and needle attached to the end of the Geomagic Touch device), including a force sensor to achieve force-profile-based haptic rendering, and (**d**) hardware systems used for our development (a Dexmo haptic glove, HoloLens 2, and Geomagic Touch, from the left).

To simulate IV needle insertion with realistic variable conditions such as human skin characteristics (e.g., color, textures, roughness), vein characteristics (e.g., shape, size, thickness, location), and different types of IV catheters, we developed a prototype of bimanual haptic simulation using nonhomogeneous but complementary haptic devices (a haptic glove and a haptic stylus, as seen in Figure 1 and Figure 2d). The bimanual haptic simulation was integrated with a mixed reality system (Microsoft HoloLens 2) that allows a user to see their real hands as well as virtual graphic images (a virtual patient and needle) when conducting the bimanual IV needle insertion procedure. In the following section, details including technology development and implemented methods are presented.

### 3.1. Development of a Bimanual Haptic Interface Integrated with Mixed Reality

The haptic mixed reality IV simulation system (HMR-IV Sim) developed in this study consists of two major components (MR graphic rendering and haptic rendering), as seen in Figure 1. For the graphic component of mixed reality, a graphic scene composed of a human patient, a vein, and the IV needle were created as a 3D mesh model (see Figure 2a,b), which were rendered in Unity using the built-in render pipeline to provide efficient low-quality forward rendering with single pass that implements only the brightest directional light per pixel for each object when there are multiple light sources. Then, the Unity built-in shader, which is a physically based shader [19], was applied to increase the realism of interactions in the graphic rendering by adjusting the following parameters: metallic: 0; smoothness: 0.5; normal map scale: 1; specular highlights and reflection enabled. In addition, graphic rendering parameters simulating variable conditions (e.g., colors and textures of the skin and veins; vein size, shape, and location; IV needle size and blood drawing) were developed and set to allow for flexibility in programing for nursing students to practice with various conditions. These parameters were synchronized with the haptic rendering to achieve a realistic simulated environment that considers both the visual and haptic perceptions expected in real-world scenarios. For example, to control the variability of vein size and location, the graphic rendering interface was programmed to be selected at 10 mm or 7 mm for the vein diameter and 5.5 mm or 7 mm for vein location (under the skin surface). These values were informed by the literature and confirmed by clinical experts (faculty members of the Kent State University (KSU) College of Nursing).

In this study, mixed reality (MR) was implemented using advanced MR glasses, namely Microsoft HoloLens 2 from Microsoft, Redmond, WA, USA (Qualcomm Snapdragon 850; 2018 × 1048 pixels per eye; 8 GB RAM, 8 MP, and 1080p camera). HoloLens 2 is a standalone system requiring synchronization with the PC-based Unity platform via WiFi through the OpenXR layer. The holograms are displayed through the HoloLens 2 by a holographic remote system that renders the image from the PC and transfers it to the HoloLens 2 in real-time via the WiFi protocol.

To create a bimanual haptic interface, two different haptic devices, a haptic glove (Dexmo: 11 degrees of freedom (DoF); 100 × 155 × 45 mm (W × H × D); 300 g; 0.5 N.m torque; Bluetooth connection) and a desktop haptic stylus (Geomagic Touch: 3DoF; 160 × 120 × 70 mm (W × H × D); USB connection; max 3.3 N) were integrated into the Unity platform using the Dexmo SDK and OpenHaptics library [20]. The local coordinate systems of the two haptic devices were synchronized in the Unity platform through a calibration process (see Section 3.2) The two haptic devices are complementary to each other, in that the haptic glove is suitable for providing a virtual grasping feeling for the user to practice left-hand (or nondominant) positioning or grasping, while the haptic stylus is able to simulate accurate IV needle insertion using the right (or dominant) hand (see Figure 2b).

For haptic rendering, we utilized an existing algorithm provided for the haptic devices in terms of basic colliders and force feedback. To create a feeling of realistic needle insertion, we developed a force-profile-based needle insertion algorithm (see Section 3.4). In the algorithm, variable conditions were implemented. However, rather than graphic variables, we focused on stiffness for the vein and skin due to its importance to creating a haptic feeling that mimics real-world experience. These stiffness parameters were implemented to be adjustable, allowing for two distinguishable values for skin and vein based on a discrimination threshold estimated by a pilot psychophysical experiment conducted to measure a human differential threshold for haptic stiffness discrimination in the presence of MR graphic objects (see Section 3.5).

The HoloLens 2 system provided a hand-tracking algorithm for hand-gesture-based interactions through the mixed reality toolkit (MRTK). However, the provided hand tracker for bare hands often failed to track the user’s hand wearing a haptic glove due to the occlusion of the fingers and hand by the mechanical parts. To resolve this issue, a Leap Motion Controller (Leap Motion Inc., depth sensor; FOV 150 × 120 degrees, 120 fps; see Figure 1) was employed to achieve accurate global hand (haptic glove) tracking in combination with the HoloLens 2 hand tracker (see global hand-tracking method described in Section 3.3).

Furthermore, in regard to the system setup, the end of the haptic stylus was modified to feature a real IVC needle (see Figure 2c). This modification allows the user to hold a real IVC needle instead of the original haptic stylus, providing a realistic grip feeling when the insertion procedure is performed. In addition, the Leap Motion Controller, tracking the left hand wearing the haptic glove, was mounted to the center (right above the camera) of the HoloLens 2, implemented after a series of pilot experiments comparing the hand-tracking performance in different locations (see Section 3.3). The hardware systems used in this study are seen in Figure 2d.

### 3.2. Calibration of the Haptic Stylus Device and HoloLens 2

Calibration of the HoloLens 2, which displays both virtual and real objects, is necessary to spatially synchronize a virtual needle with a real needle attached to the haptic stylus device in motion. Without calibration, there would be discrepancies in position and direction between the virtual and real needles, leading to misperceptions of the user’s needle insertion task in the mixed reality system. Especially for this study, accuracy of the calibration is critical, since simulated IV needle insertion requires hand–eye coordination as well as fine hand/finger motor skill in terms of locating and inserting the thin virtual needle. The HoloLens coordinate system is a right-handed coordinate system that enables the ability to track the HMD position in the real world. Once the user can define a stage that represents the room in the real world, the stage defines a stage origin, which is a spatial coordinate system centered at the user’s position and orientation. The HMD position is tracked based on the stage origin. For this reason, a consistent world coordinate system is needed for calibration within the virtual world, and haptic coordinates are needed to simulate the needle insertion with an overlayed syringe on the haptic stylus.

**Figure 3:**
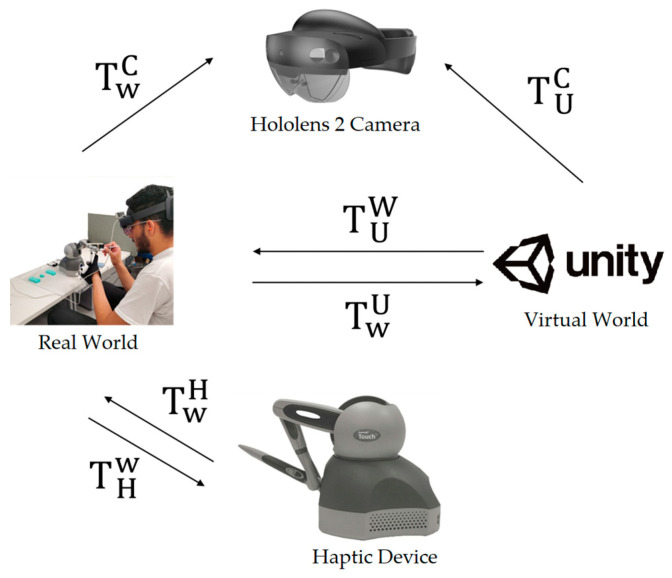
HoloLens 2 camera calibration with the haptic stylus system in the real world, and the relationships between the real world (3D) and the virtual world (Unity 3D), including the HoloLens 2 camera. The transformation matrices were computed by our calibration process.

Our calibration method relies on the initial positions of the HoloLens 2 camera and the haptic stylus device, both measured in the real-world coordinate system. To fix the initial positions of the HoloLens 2 and the haptic stylus, we designed a device-positioning board (an acrylic panel (width: 41 cm; height: 26.5 cm; depth: 26.5 cm) with positioning guides for the HoloLens 2 and haptic stylus to be positioned at predefined positions when our system starts. In this way, the synchronization of different coordinate systems was well maintained, even when the HoloLens device was dynamically in use by the user. Calibration data computed through this process are prestored and automatically loaded when the HMR-IV Sim system starts, as shown in Figure 1. To implement the calibration, our approach takes two steps: HoloLens 2 camera calibration with the haptic stylus and coordinate system synchronization between the real world (3D) and virtual world (Unity 3D). By taking these two steps, the real IVC needle in the haptic local coordinate (3D) is transformed to the real-world coordinate (3D) system through the coordinate system synchronization process and then projected to the HoloLens camera by a projection matrix obtained by HoloLens camera calibration. The relationship between transformation matrices is demonstrated in Figure 3.

To estimate an accurate transform operation from the coordinate system of the haptic device to the eye view of the HoloLens 2, we propose an MR camera calibration method using Direct Linear Transform (DLT) [21] combined with coordinate system synchronization. Due to the offset between the HoloLens 2 camera position and both eye view positions, the holographic object is overlapped onto the real object in the eye view, and the HoloLens 2 camera view shows the gap between the holographic and the real object. The root mean square error of the gap from all real control points to virtual control points on the projected image is (−24.5, −31.5) pixels, (−20.9, −21.1) pixels, and (−24.1, −28.25) pixels for captured image from 0, −45, and 45 degrees, respectively.

The DLT-based calibration method requires at least 6 control points as pairs of corresponding points between 3D (*x,y,z*) and 2D image coordinates (*u,v*). The control points, which are 3D points in the real-world coordinate system and projected pixel points on the camera image plane, are obtained by measurements and then used for estimating the unknown parameters (called DLT parameters) of a 3 by 4 homogenous camera matrix *P* by solving linear equations ([*u; v; w*] =*P* * [*X; Y; Z; W*]). The projection matrix *P*, consisting of the estimated parameters, maps any 3D points to the camera image plane, which becomes the HoloLens 2 camera view.

In this study, a checkerboard box was designed for HoloLens camera calibration using the DLT method. The calibration box is covered with a checkerboard (each square side: 1 cm). Each side of the box contains 4 control points at each corner of rectangle to place the point far from other points and cover the calibration space of the box. For calibration, a total of 12 control points at 3 different sides of the box were selected. The positions of control points in the real-world coordinate system and the corresponding pixel points on the image plane of the HoloLens camera were measured using a ruler and an image viewer tool (Paint), respectively. The gyro sensor was used to maintain the position and orientation of HoloLens camera in this step. The origin of the real-world coordinate system is the left front corner of the synchronization board, and for the axis, we used the right-handed coordinate system. The control points were then used for computing an initial projection matrix using the DLT method. In this step, 2 points from each side, or a total of 6 points, were selected to create the matrices and select the matrix with minimum error as the initial matrix. Then, the initial matrix was iteratively optimized using Broyden–Flether–Goldfarb–Shanno optimization (BFGS) algorithm [22], which finds the minimum reprojection errors in the camera image coordinates. As illustrated in Figure 3, this calibration process was repeated for estimating two projection matrices, $T_{W}^{C}$ (the real world to the HoloLens camera for the synchronization of the real needle) and $T_{U}^{C}$ (the virtual world (Unity) to the HoloLens camera for the synchronization of the virtual needle).

The next step of the calibration process is computing the homogeneous transformation matrices between 3D coordinate systems, namely the real world, the virtual world (Unity), and the haptic stylus system, as each has a different coordinate system. For the coordinate synchronization, four (4 × 4) transformation matrices ($T_{W}^{H}$, $T_{H}^{W}$, $T_{U}^{W},\;T_{W}^{U}$) were computed using 12 corresponding points between paired coordinate systems (e.g., the real world to the virtual world, the virtual world to the haptic stylus, the real world to the haptic stylus), and then matrices were optimized with the BFGS. The cost function of the BFGS is the root mean square error of all 12 points generated by 12 4 × 4 homogeneous matrix parameters.

### 3.3. Global Haptic Glove Tracking to Improve Touch Interaction

Most ungrounded haptic gloves are not capable of tracking the user’s hands or fingers globally in the world coordinate system but provide local finger tracking referencing the center of the palm of the glove. In this study, we used an ungrounded Dexmo haptic glove [23], one of the most advanced commercially available haptic gloves, so the user could grasp the hand of the virtual patient displayed through the MR headset using his/her left hand, which requires global motion-based gestures.

The lack of global tracking function of the haptic gloves led us to consider the built-in hand tracking of the HoloLens 2 depth camera. However, it was learned through a pilot experiment with the HoloLens 2 system that the built-in hand tracking was optimized for bare hands and often failed to track a hand wearing an exoskeleton haptic glove due to mechanical links and parts occluding the user’s hand. To improve the global hand-tracking performance of the HoloLens 2 system, we combined it with a Leap Motion Controller device, an external depth sensor performing best at bare-hand tracking at a close distance (less than 60 cm). We then developed and combined a hand-tracking algorithm that automatically selects either sensor (Leap Motion or HoloLens 2) to accurately obtain tracking in a dynamic mixed reality scene. The algorithm placed a higher priority on the Leap Motion because it demonstrated better performance tracking the haptic glove (facing down) while in motion (see Table 1). For this tracking algorithm, synchronization of the two coordinate systems (Leap Motion and HoloLens 2) is required first so that hand-tracking data from the two different sensors can be aligned in a single coordinate system. To achieve this, the same method of coordinate system synchronization described in Section 3.2 was applied, using 12 control points sampled from both coordinate systems for estimating a 4 by 4 transformation matrix that maps the Leap Motion sensor coordinates to the HoloLens 2 coordinates with a minimum error (RMSE: 8.6 mm).

It was also learned that the hand-tracking performance was significantly affected by illumination and camera viewing angles to be determined by the location of a depth sensor. We therefore conducted a pilot experiment to find the best location of the Leap Motion sensor for two mounting scenarios (on the desk or head (HoloLens 2)), comparing the success rates of tracking a haptic glove that was worn and tested with different hand postures and gestures (facing up or down, grasping a virtual hand) using single-sensor-based methods or our method. The success rate of hand tracking was computed using the metric $SR=\frac{t}{n}$, where SR denotes the success rate of hand tracking, *t* denotes the frames during which the glove is tracked, and *n* denotes total frames.

The experimental results (Table 1) suggest the center of the MR headset (on the head) was the best location, and it was adopted in this study. The mounting location was determined by measuring the accuracy of hand tracking combined with HoloLens 2 while considering the ergonomics of the user’s head in motion. No subjects reported discomfort with the Leap Motion mounting. It also confirmed that our global hand-tracking method outperforms single-sensor-based tracking methods. Although the success rate of the grasping gesture tracking is not yet optimal, it was sufficient in our study to achieve realistic haptic grasping feedback, since (1) almost all of tracking failure frames in the grasping gesture were found in the middle of the motion (not near the end, where haptic force feedback is computed) and (2) our system was developed to activate the local finger tracking of the haptic glove to compute accurate force feedback once the user’s hand has been successfully located at the desired grasping position. In this way, the global hand tracking of the haptic glove was significantly improved. Our global hand-tracking algorithm was designed to select either hand tracker (HoloLens 2 or Leap Motion) based on each tracker’s tracking status (failure or success). The virtual hand (palm center) position of the haptic glove is updated with the tracking information of the selected tracker. If both trackers fail to track the haptic glove, our algorithm holds the virtual hand at the last updated position.

### 3.4. Haptic Rendering for IV Needle Insertion and Virtual Grasping

In this study, two haptic rendering schemes were developed to simulate realistic IVC needle insertion using the modified 3DOF haptic stylus (see Figure 2c) and grasping a virtual hand or arm using an 11 DOF exoskeleton glove (see Figure 2d). For the IVC needle insertion haptic rendering, we created a force-profile-based haptic rendering algorithm. The algorithm consists of two parts. First, we created a multilayer mesh-based force-rendering framework optimized for simulating IVC needle insertion with adjustable parameters (stiffness and friction). Second, we determined the optimum values of the adjustable parameters using force-profile-based data analysis and expert feedback.

**Figure 4:**
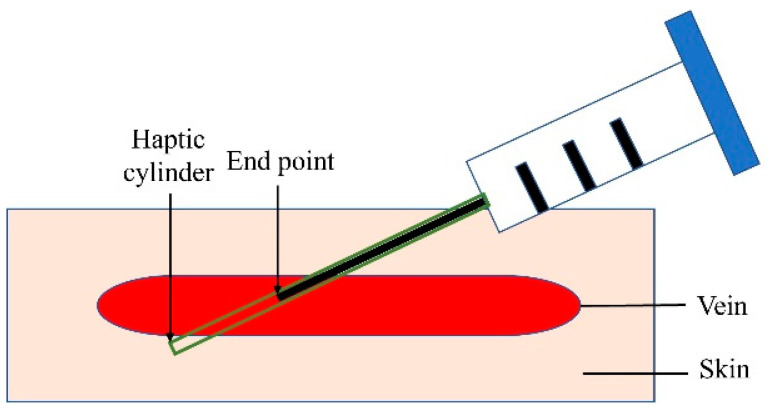
A multilayer mesh-based force rendering developed in this study. When needle insertion occurs, the haptic cylinder mesh object is automatically created and attached to the virtual needle to guide an insertion path determined by an initial insertion angle on the skin surface.

The multilayer mesh-based framework uses multiple 3D mesh objects (skin, vein, needle) at different layers to create different haptic feelings at the skin and vein layers, respectively, as graphically illustrated in Figure 4. Each mesh object was designed to have its own customized mesh collider that detects the collision of the virtual needle accurately. In addition, for the inserted needle to stay at one spot on the skin surface, we added a cylinder-shaped mesh object (Haptic cylinder in Figure 4) to the virtual needle, which haptically guides a penetration path determined by the initial insertion angle on the skin and mimics a realistic insertion feeling created by the inner layer of the skin. The resisting force of the haptic cylinder object is computed only when the end point of virtual needle is moved into the skin surface, which is detected by the position sensor of Geomagic Touch (3D Systems Inc., Santa Clarita Valencia, CA, USA). The resisting force is optimized by stiffness and friction. Once the haptic cylinder is activated, the needle is not able to break the collider of the cylinder until the end of insertion. Insertion feedback forces were computed using the virtual proxy model [24,25] implemented in the OpenHaptics library with adjustments to provided haptic rendering parameters (stiffness, friction, pop-through).

**Figure 5:**
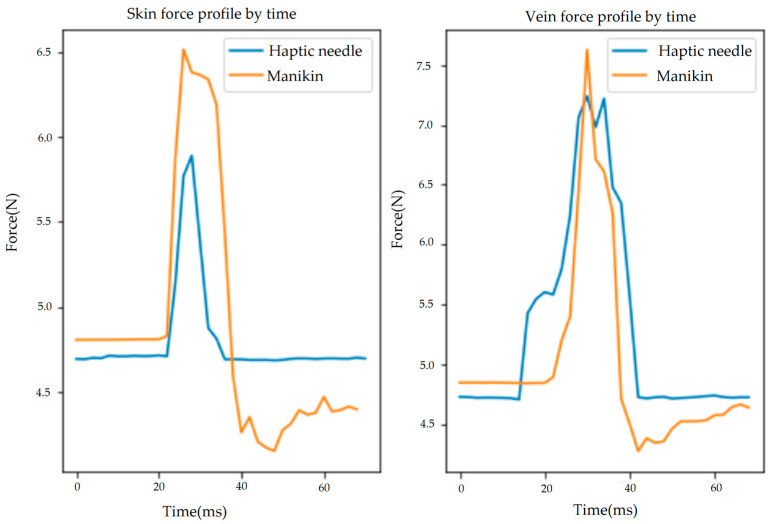
Force profiles recorded with a manikin arm and our haptic needle insertion simulation system for the skin and vein, respectively. These pairs exhibit the best similarity results, proven by the highest correlation coefficients (**Left**: 0.9511; **Right**: 0.8762). Although the gap between the two force profiles (insertion into a virtual vein using the haptic needle vs. insertion into a real manikin arm) looks large in such a high-definition force dimension, it was perceptually acceptable and confirmed by experts (nursing faculty) as the most realistic insertion feeling.

The second step was to determine the optimum values of haptic parameters when inserting into the skin or vein. To objectively complete this process, force-profiling-based data analysis was conducted. Our approach utilized a clinical expert’s force profile as the reference point to be similarly formed by the multilayer mesh-based force rendering. Through this process, we focused on estimating the optimum value of stiffness that causes a realistic haptic feeling for the skin and vein. Force profiling with a nursing faculty member (Jeremy Jarzembak) was conducted using a high-precision force sensor (Load Cell Systems, Towanda, PA, USA, MCL-01-50N) attached to a real IVC needle during insertion into a manikin arm (VEVOR Intravenous Practice Arm), commonly used in nursing schools. Two force profiles (orange curves in Figure 5) were recorded for the skin and vein, respectively. It is obvious that the peaks and shapes of the two profiles are different—the larger and sharper force peak formed in the vein. To find the best value of stiffness, the same force profiling was conducted using the same force sensor attached to the modified haptic stylus (see Figure 2c) of our haptic simulation system. This force profiling was repeated while changing the value of stiffness until sufficient force samples were collected to be iteratively compared with the two-reference profile. All the profiles were recorded for 0.2 s and then synchronized and compared using the Pearson correlation method [26]. Figure 5 shows the comparisons and the best correlation values (similarity: 0.9511 and 0.8762 for the vein and skin, respectively), which determine 0.5 and 0.8 as the best values of stiffness for the vein and skin, respectively. This value was also confirmed by the expert. Other minor parameters were also optimized by the expert’s feedback. The final optimum values of haptic parameters used for the haptic needle simulation are as follows:

- Skin: stiffness 0.8, damping 0.9, static friction 0.2, dynamic friction 0.2, pop-through 0.02.
- Vein: stiffness 0.5, damping 0.9, static friction 0.2, dynamic friction 0.3, pop-through 0.057.

Our haptic glove rendering was implemented using both OpenHaptics [20] and Dexmo SDK in Unity to simulate grasping a virtual hand while a virtual needle is inserted. As mentioned above, global hand tracking described in Section 3.3 was combined with the haptic finger tracking function provided by the Dexmo SDK. Resisting forces (max 0.5 N) were computed when the virtual fingers of the haptic glove touched the virtual hand surface for grasping. This force computing was implemented using the virtual proxy model [20].

### 3.5. Perception-Based Haptic Variability Rendering

Rather than visual variables (color, textures, size, shape, etc.), stiffness (hardness) determines the haptic variability of the skin and vein. However, a change in stiffness is invisible and completely unknown until the user feels it using a haptic device. Since we replaced the end part of the haptic stylus with a real IV needle, it was necessary to find a discrimination threshold of haptic stiffness when using a real needle in the context of fully immersive mixed reality. We therefore conducted a pilot experiment to estimate a discrimination threshold using the method of limits [24], one of the classic psychophysical methods. The reason we chose the method of limits is that it estimates a perception threshold efficiently and quickly, though with relatively lower accurately, which is still sufficient to determine two distinguishable values of stiffness for our system. For the pilot perception study, four participants (average age 24.5, 3 males and 1 female) took part in the experiment. During the experiment, participants were asked to wear the HoloLens 2 glasses and touch/feel the surfaces of two virtual cubes (one reference (value: 0.5) and one test stimuli (a value in the range of 0–1)) using the real needle interface attached to the haptic stylus device. Ascending and descending series were presented alternately 10 times each. The step size of stimulus increments, or decrements, was 0.1. For each trial, participants were asked to answer which one (cube) was stronger. The estimated discrimination threshold from this pilot study is 0.169 ± 0.021. With this result, we determined two distinguishable values (skin stiffness: 0.8 and 0.63; vein stiffness: 0.5 and 0.33) that were also verified by the expert. These values were applied to the haptic IV needle rendering (Section 3.4 and Figure 1).

## 4. Evaluation Study for Measuring Usability

An evaluation experiment was conducted to measure the usability of the HMR IV needle simulation system with human subjects from novices to experts for an IVC needle insertion task using two hands.

### 4.1. Participants

Twenty healthy participants (14 males, 6 females) with an average age of 32.4, all of whom were right-handed, were recruited from the College of Nursing or College of Medicine and voluntarily took part in the experiment. Nine of them were experts who had formal training and had performed more than five successful IV insertions with real patients, while eleven were novices who had no formal IV training or insertions. Each participant was paid $10 per hour and provided a written consent form approved by the Kent State University institutional review board (IRB).

### 4.2. Apparatus

**Figure 6:**
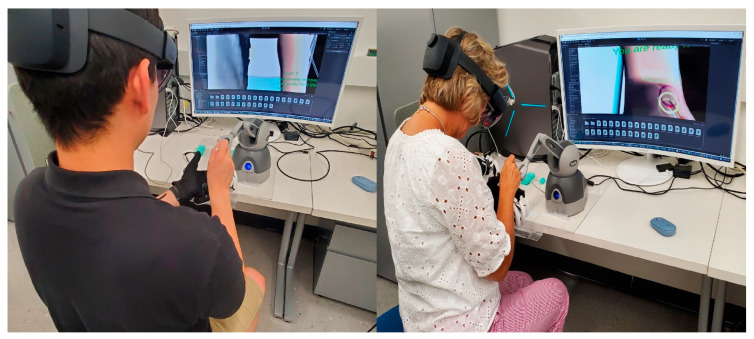
Participants performing the IV needle insertion procedure (**Left**: an expert (a faculty member of the KSU College of Nursing); **Right**: a novice).

The HMR-IV insertion simulation system developed with a 64-bit Windows desktop PC (Intel^®^ core™ i7-9900K CPU from Intel, Santa Clara, CA, USA, 32 G RAM, and a NVIDIA RTX 2070), a Geomegic Touch haptic device (right hand), a Dexmo haptic glove (left hand), and Microsoft HoloLens 2 were used for this evaluation study, as seen in Figure 6. A 32” PC monitor was used only for monitoring the experiment.

### 4.3. Procedure

When participants arrived, they were asked to sit in front of a desk where the simulation devices (haptic devices and a HoloLens 2 system) were placed. Participants were given time to sufficiently familiarize themselves with the IVC needle insertion system after learning the usage protocol. For novice participants, a short tutorial about practicing IVC needle insertion (grip, insertion angles, a pop feeling in the vein, etc.) was also provided. In the main experiment, participants were asked to repeat an IV needle insertion 64 times (trials) with different variabilities. For each trial, variability conditions (vein location, vein size (diameter), haptic skin, and vein stiffness, with 2 distinguishable levels each) were randomized. Participants were also asked to use earplugs to block external noise. For each trial, instruction texts (start, end, actions to take, etc.) were sequentially displayed through the HoloLens display for participants to complete the experiment without assistance. To guide the target touch positions with haptic devices, visual graphic feedback was provided. For the haptic glove, a semitransparent hand image overlaid on the haptic glove turned green from red, and for the IV needle, a target insertion area was highlighted by an oval. For each trial, participants were asked to wait for 5 s without motion when they were confident about a successful insertion into the virtual vein. After completing the main experiment, participants were asked to fill out a questionnaire and the NASA Task Load Index (TLX). The questionnaire includes questions such as these: (1) Did you find the haptic feedback useful when performing the needle insertion? (2) Did you find this exercise more educational than performing on a physical manikin model? (3) Do you think this experience was of value to your training? (4) Would you like to continue using this type of training experience in the future? It took on average 35 min for each participant to complete the experiment.

### 4.4. Data Analysis

To measure the usability of the IV needle insertion simulation system, we conducted both quantitative and qualitative data analyses. For quantitative data analysis of success rates of needle insertion, the insertion angles (5 to 30 degrees), task completion time (start and end), and distance (the needle tip end to the vein center) were measured by functions developed in our system. A success rate was calculated by a formula: the number of successful insertions divided by total attempts. The requirements of a successful insertion, defined by the expert using evidence-based practices set forth by the Infusion Nurses Society, include (1) an insertion angle between 5 to 30 degrees (ideally 20 degrees) and (2) the needle tip staying inside the vein once inserted. For qualitative data analysis, subjective responses to both the questionnaire and NASA TLX were analyzed to measure the usability of our system.

## 5. Experimental Results

### 5.1. Results: HMR IV Needle Simulation System Verification and Calibration

To verify the real-time computing performance of the developed system, the update rate of the entire system was measured while the haptic system was running at an update rate over 1 Khz, which is the minimum requirement for real-time haptic rendering. For the update rate measurement, total frames were divided by the elapsed time from needle insertion start on the skin to the end. This was repeated 10 times and then averaged. The result was 56 frames per second (0.018 s per frame), which is sufficiently fast to conduct the bimanual IV needle insertion procedure in real-time.

**Figure 7:**
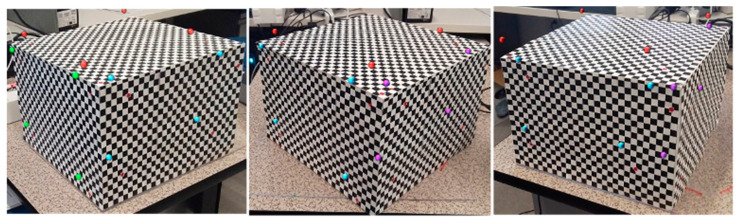
Calibration checkerboard box with virtual feature points captured by HoloLens 2 camera at three different viewing angles (−45, 0, 45 degrees). Each color of the virtual feature points demonstrates the point from same side of the box.

The system calibration described in Section 3.2 was conducted to compute all the transformation matrices illustrated in Figure 3. For the calibration of the HoloLens 2 camera, a checkerboard box (Figure 7) was designed and used for sampling and measuring control points in the real and virtual worlds. To collect control points from 3 different sides, the box was placed tilted diagonally, while the viewing angle was 0 degrees. Calibration errors, determined using reprojected pixel points by the estimated camera projection matrix, were calculated at three different viewing angles (HoloLens 2 viewing direction: −45 degrees (left), 0 degrees (front), 45 degrees (right)) on the horizontal axis to cover possible viewing scenarios during the experiment. In addition, all other errors of the estimated transformation matrices between 3D coordinate systems (real world, virtual world (Unity), and haptic device local coordinates) were also calculated using 3D reprojection points. Table 2 summarizes the errors (root mean square error (RMSE) computed). The inverse direction of calibration generated the inverse of the matrix. Therefore, only one-way RMSE is provided in the table. The overall results are sufficiently accurate to implement well-synchronized hand–eye coordination using our simulation system, although four different coordinate systems (virtual world (Unity), real world, haptic world, and HoloLens-based mixed reality world) were integrated to achieve mixed-reality-based fine motor skill training. The average calibration error $T_{W}^{C}$ is 3.56 pixels. This error shows the needle registration error in the eye view is less than 1 cm during the needle insertion process. If the headset was more than 5 m from the working space, the error was distinguishable. However, if the needle insertion task proceeded in a small working space, the error did not increase significantly. In the experiment, participants adapted to the system easily until they started the main experiment.

### 5.2. Results: Evaluation Study of Measuring Usability

Quantitative and qualitative data obtained through the evaluation user study were thoroughly analyzed, and results, including statistical analysis, are reported in this section.

The quantitative results are based on measurements (success rate, completion time, distance from the needle tip to the vein center, insertion angle) automatically recorded in our developed system during the experiment. We compared the data between two groups (novice and expert) and further conducted statistical analysis using a *t*-test to see if there is any significant difference between the two groups.

**Figure 8:**
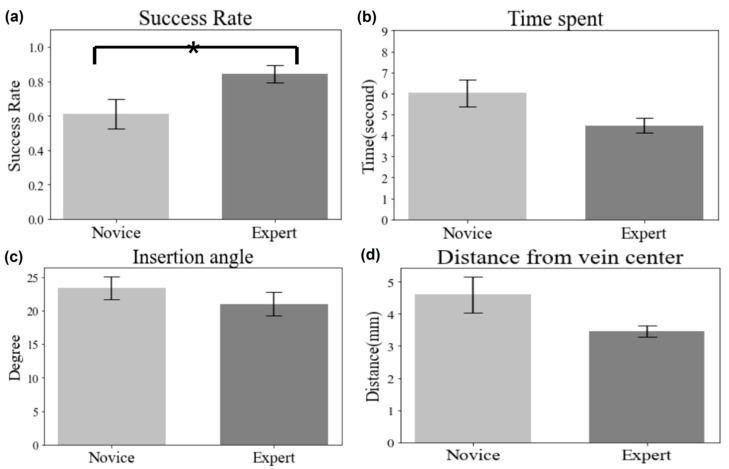
Results of user performance measurements: success rate, task completion time, insertion angle, and distance between the vein center and the last position of the virtual needle tip, compared between novice and expert groups. “*” is displayed on the chart when *p*-value from *t*-test is lower than 0.05 (* *p* < 0.05).

Figure 8 compares the quantitative results between two groups (novice and expert) for all trials regardless of variabilities. The results show that experts succeeded in more trials than novices, which is also confirmed by *t*-test (Novice: 0.61 ± 0.09%; Expert: 0.84 ± 0.05%; *p* = 0.035). It was determined by *t*-test analysis that the experts took less time to complete the needle insertion procedure (Novice: 6.02 ± 0.64 s; Expert: 4.47 ± 0.34 s; *p* = 0.029). Regarding the insertion angle, the expert group performed better than the novice group, demonstrating a smaller gap from the desired angle, but no significant difference was detected (Novice: 23.39 ± 1.73 degrees; Expert: 21.03 ± 1.74 degrees, *p* = 0.176). For novices, the needle tip’s location relative to the center of the vein was noted to be further away compared to the experts’ performance, which was significantly different (Novice: 4.6 ± 0.6 mm; Expert: 3.4 ± 0.16 mm; *p* = 0.046).

**Figure 9:**
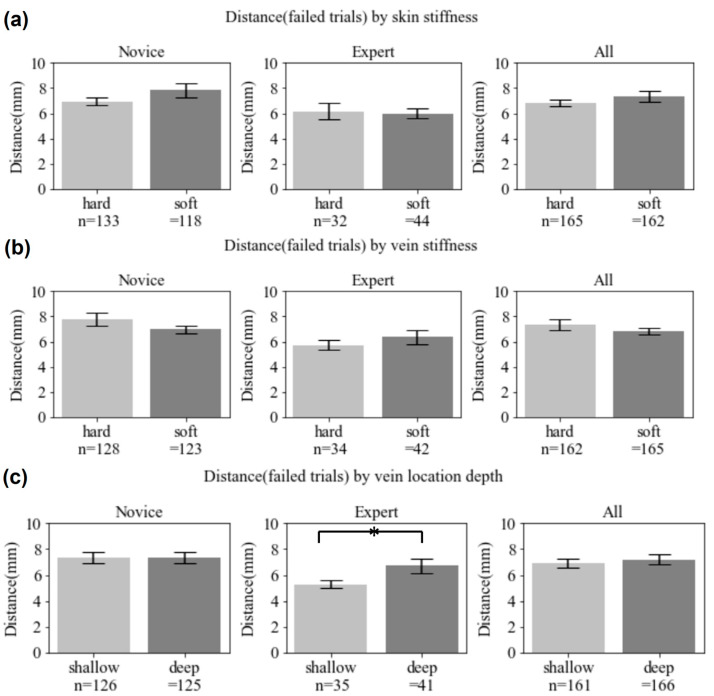
Analytic data comparing failure trials in terms of two variabilities (haptic stiffness for the skin and vein and vein location depth). For each figure, *n* is the failed attempts, and the vertical axis is the distance measured from the vein center to the needle end tip (* *p* < 0.05).

Figure 9 shows analysis regarding variabilities (haptic stiffness and vein location depth), which provides an understanding of how the variabilities were well designed to control insertion difficulty levels in our system. Our focus was analyzing failed attempts associated with two difficulty levels and the variabilities described in Section 3: haptic distinguishable stiffness (soft and hard for the skin and vein, respectively) and vein location depth (shallow and deep). Distance measurements (needle end tip to the vein center) were also compared to see which group’s performance was better, even for failed attempts. In terms of haptic stiffness, results were not consistent between groups. The novice group demonstrated difficulty with the harder surface for insertion into both the skin (133 vs. 118) and vein (128 vs. 123), while the expert group showed the opposite (32 vs. 44; 34 vs. 42). Results of the expert group were consistent for both the skin and vein. Regarding vein location depth, both groups had difficulties with the vein location further from the surface. This difference becomes clearer in the expert group, confirmed by statistical analysis of the distance (Expert: Shallow = 5.31 ± 0.26 mm; Deep = 6.72 ± 0.58 mm; *p* = 0.041).

Table 3 shows the results (success rate, completion time, and distance to the vein center) versus the other variability (vein diameter: big and small). Based on the results of Table 3, it is obvious that the participants’ performance was significantly affected by the variability, which was also confirmed by further statistical analyses for both groups, but only the novice group was significantly affected by the vein diameter condition, and this was the case for all the metrics: success rate, completion time, and the distance to the vein center in failed trials.

**Figure 10:**
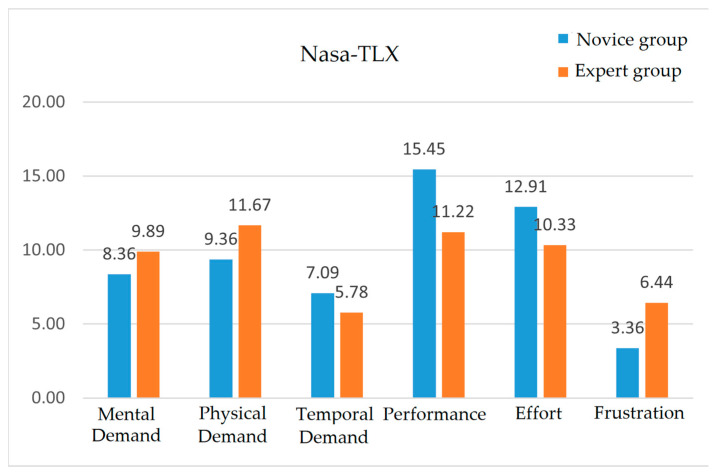
Results of workload measurements by the NASA TLX [27].

To analyze the user’s perceived workload while using our developed system, rates (0 (low) to 21 (high)) of the six questions addressed by the participants were analyzed. The definition of each category can be seen in Table 4. Figure 10 shows that the highest demand was Performance in both groups, while the lowest was Frustration or Temporal Demand. For the other questions, all participants in both groups responded “yes” to Questions #1 (haptic feedback is useful) and #4 (would like to continue using this system). For Question #2 (this system is educational), all participants responded “yes” except for one novice participant. For Question #3 (value of our system for training), all participants except one expert participant responded “yes”.

## 6. Discussion

IV insertion is a technically difficult procedure to master, as demonstrated by the 35–50% failure rate in real clinical environments [28] where nursing professionals are normally experiencing various conditions. The results in Figure 8, Figure 9 and Figure 10 show how well our developed system provides a bimanual virtual practice environment simulating a realistic environment with variable insertion conditions. Figure 8 shows a clear distinction in needle insertion skills between novices and experts in terms of success and accuracy. To implement variable insertion conditions, which should improve the limited exposure to rare or unexpected conditions when practicing, the overall success rates consistently show the performance of expert group decreased with increased difficulty level of each variable condition (see Figure 9 and Figure 10). Changes in the experts’ performance are obvious within all the variable conditions (vein depth location and vein diameter), but haptic stiffness (skin and vein) will require further investigation and analysis. Haptic stiffness could be due to the inaccurate discrimination threshold roughly estimated by the pilot study. The other rationale could be that two values (chosen for vein or skin stiffness) are too close to each other to be distinguishable. This will be further investigated in a future study.

Finally, the NASA TLX results inform us that the system is developed well enough for both groups (novices and experts) to use it for practicing the needle insertion procedure despite the complexity of combining two haptic devices with MR glasses. The highest success rate being for Performance and lowest rate being for Frustration demonstrates the easiness and comfortableness of the HMR-IV simulation system despite it presenting a mentally and physically high demanding task. The biggest difference between the two groups being observed in Frustration is possibly due to the age gap of the two groups—almost all experts were older than young novices, who are fast adapters to new technologies.

The system’s limitation was its low rate of tracking the grasping gesture (47.81%), which caused freezing for the global position of the glove while the participant attempted to grasp the virtual patient’s hand. Once the glove tracking system loses the glove position, it takes a long time to recover the glove detection. To enhance glove tracking performance, we will consider new approaches such as attaching trackers to the glove or creating algorithms aimed at glove tracking in future work. Once we verify the usability of the enhanced system, our research will proceed to identify the learning effect of the system compared to the traditional method in schools and colleges of nursing.

## 7. Conclusions

IVC needle insertion is not an easy skill to master due to lack of clinical practice with real patients while in nursing school. Current state-of-the-art technologies lag behind what is needed to develop psychomotor skills that mimic real-life clinical practice with variable insertion conditions, bimanual hand–eye coordination with realistic tactile feelings, customizable self-paced learning, unlimited access, and immersive engagement. In this study, we developed a bimanual haptic interface system using two different haptic devices (a glove and a stylus) to accurately simulate the realistic insertion procedure, interacting with a virtual patient displayed through a mixed reality headset (HoloLens 2). Our system provides a variability setting function for customizing variable insertion conditions (color, texture, haptic stiffness and friction, and geometric properties of the skin and vein), considering real clinical environments and objective performance measurements and monitoring insertion angles, distance, and trajectory for self-paced learning. Evaluation results with human subjects support an educational use at schools or clinics, which suggests a promising direction for this research. However, this initial study should be confirmed by conducting a full training study to measure the learning impact of the current system, which will be conducted in the near future. Therefore, in the future, we will advance the current force-profile-based haptic rendering by replacing the current (manikin arm) force data with real human data. We will also investigate the effect of this system on the learning performance of nursing students in a classroom setting by adding more variabilities and realism.

## Figures and Tables

**Table 1:** Successful tracking rates of global hand tracking with a haptic glove for different hand postures and mounting positions of the Leap Motion device.

Haptic Glove Worn	Hand Postures (No Motion)		Hand Gesture (Motion)
Sensors	Facing up (Palmar surface)	Facing down (Dorsal surface)	Grasping a virtual hand (Mixed surface)
HoloLens 2	0.9969	0.0016	0.4781
Leap Motion (on desk)	0.0867	0.9454	0.0587
Combined (on desk)	0.9969	0.9454	0.4781
HoloLens 2	0.9457	0.0087	0.4939
Leap Motion (on head)	1	0.6474	0.5215
Combined (on head)	1	0.6474	0.6364

**Table 2:** Calibration Errors (Root mean square).

	Left (−45 Degree)	Front (0 Degree)	Right (45 Degree)	Average
$T_{U}^{C}$	0.35 pixels	0.78 pixels	0.7 pixels	0.61 pixels
$T_{W}^{C}$	3.63 pixels	4.02 pixels	3.03 pixels	3.56 pixels
$T_{U}^{W}$	1.87 cm	1.64 cm	2.63 cm	2.04 cm
$T_{H}^{W}$	0.9 mm			

**Table 3:** Success rate, completion time, and distance from vein depending on the sizes of vein diameters in each group.

	Novice	Expert	All
Success rate	Big: 0.74 ± 0.08	Big: 0.93 ± 0.04	Big: 0.83 ± 0.05
	Small: 0.48 ± 0.1	Small: 0.75 ± 0.07	Small: 0.6 ± 0.069
	*p*-value = 0.057	*p*-value = 0.038	*p*-value = 0.014
Completion time	Big: 5.52 ± 0.2 s	Big: 4.02 ± 0.14 s	Big: 4.84 ± 0.13 s
	Small: 6.54 ± 0.23 s	Small: 4.92 ± 0.15 s	Small: 5.81 ± 0.15 s
	*p*-value = 0.001	*p*-value < 0.001	*p*-value < 0.001
Distance from vein: fail trials	Big: 8.62 ± 0.59 mm	Big: 8.76 ± 1 mm	Big: 8.64 ± 0.56 mm
	Small: 5.71 ± 0.28 mm	Small: 5.71 ± 0.28 mm	Small: 6.5 ± 0.26 mm
	*p*-value = 0.005	*p*-value = 0.004	*p*-value < 0.001

**Table 4:** Nasa TLX six categories.

Nasa TLX Questions
[Mental Demand] How mentally demanding was the task?
[Physical Demand] How physically demanding was the task?
[Temporal Demand] How hurried or rushed was the pace of the task?
[Performance] How successful were you in accomplishing what you were asked to do?
[Effort] How hard did you have to work to accomplish your level of performance?
[Frustration] How insecure, discouraged, imitated, stressed, and annoyed were you?

## Data Availability

The data presented in this study are available on request from the corresponding author.
